# Incidence and associated factors of congenital syphilis at a tertiary care center in Thailand

**DOI:** 10.2478/abm-2023-0039

**Published:** 2023-08-07

**Authors:** Pimchanok Kulsirichawaroj, Dissajee Lumbiganon

**Affiliations:** Department of Pediatrics, Faculty of Medicine, Vajira Hospital, Navamindradhiraj University, Bangkok 10330, Thailand; Division of Neonatology, Department of Pediatrics, Faculty of Medicine Vajira Hospital, Navamindradhiraj University, Bangkok 10330, Thailand

**Keywords:** associated factors, congenital syphilis, inadequate maternal treatment, neonatal outcomes

## Abstract

**Background:**

The incidence of congenital syphilis is increasing worldwide, in parallel with the increase of syphilis in the general population.

**Objectives:**

This study aimed to determine the incidence and risk factors for congenital syphilis at a referral tertiary care center in Bangkok, Thailand.

**Methods:**

This is a case–control study using the hospital medical records of neonates born at our hospital, whose mothers had confirmed syphilis during pregnancy or at delivery between 2011 and 2018. Maternal and neonatal data were reviewed. Neonates were categorized into congenital syphilis according to CDC surveillance case definition for congenital syphilis 2015 and the American Academy of Pediatrics Congenital Syphilis 2018: confirmed and probable were assigned to the case group, while possible and less likely congenital syphilis were used as the control group. Factors associated with congenital syphilis were analyzed using univariable and multivariable analysis.

**Results:**

Among 19,558 live births, there were 126 neonates born to mothers with syphilis. Almost 40% of mothers were teenage mothers and 48.4% had inadequate or no syphilis treatment. Forty neonates met the criteria of congenital syphilis giving the incidence of 204 per 100,000 live births (95% confidence interval [CI]: 146–278). Factors associated with congenital syphilis were inadequate treatment of maternal syphilis and preterm birth (adjusted odd ratio [aOR]: 2.69, 95% CI: 1.02–7.11, *P* = 0.046 and aOR: 2.91; 95% CI: 1.01–8.39, *P* = 0.049, respectively).

**Conclusions:**

The incidence of congenital syphilis in our institution was high. Factors associated with congenital syphilis were inadequate treatment of maternal syphilis and preterm birth. Improvement of prenatal care should be emphasized.

Syphilis is a sexually transmitted disease (STD) caused by infection with *Treponema pallidum*. It is transmitted from the mother to the child through the placenta or during birth. Infection with syphilis during pregnancy causes high obstetric and perinatal complications [1]. Complications associated with syphilis during pregnancy can be prevented by syphilis screening in pregnant women and prompt treatment with penicillin, a low-cost and highly effective drug for syphilis [2]. This has led the World Health Organization (WHO) [3] and partners to launch the global initiative to eliminate vertical transmission of syphilis in 2007 and set a goal for the elimination of mother- to child-transmission of syphilis to 50 cases per 100,000 live births by 2030.

Although syphilis infection is easily diagnosed and treatable, rates of syphilis continue to increase among the selected population globally and consequently increasing the rate of congenital syphilis [4, 5]. In the United States, the number of reported congenital syphilis cases has increased from 8.4 cases per 100,00 live births in 2012 to 33.1 cases per 100,00 live births in 2018, reaching a 20-year high [6]. A cross-sectional study from a city in Brazil found that the incidence of congenital syphilis increased from 121 to 223 per 100,000 live births in 2011 and 2014, respectively [7]. Reports on risk factors associated with congenital syphilis from the United States and other countries included lack of prenatal care, lack of antenatal screening, delayed diagnosis of syphilis in pregnant women, untreated or inadequately treated pregnant women, and low socioeconomic status [7, 8, 9, 10].

Since 2001, Thailand has been working on preventing of mother-to-child transmission of congenital syphilis along with prevention of transmission of human immunodeficiency virus (HIV) from mother to child. A monitoring system for the prevention of mother-to-child transmission of HIV (Perinatal HIV Intervention Monitoring System [PHIMS]) has been developed. In 2012, the Thailand Department of Health has started collecting data on syphilis screening in pregnant women, treatment of pregnant women and babies born to mothers infected with syphilis, along with the collection of HIV data among pregnant women in the PHIMS program [11]. In 2016, Thailand was validated from WHO for the achievement of this goal of elimination of mother-to child-transmission of syphilis with the rate of 10.9 per 100,000 live births [12]. Recently the Ministry of Public Health of Thailand reported an increase in the rate of syphilis in the general population, especially in the age group of 15–24 years paralleled with the increase in incidence of congenital syphilis from 15 to 40 cases per 100,000 live births in 2017 and 2018, respectively [13].

The increasing incidence of congenital syphilis cases is a public health concern for Thailand, but data on congenital syphilis diagnosed by using the Centers for Disease Control (CDC) surveillance case definition [14] and its associated factors are scarce. Thus, we performed this observational study to assess the incidence of congenital syphilis between January 2011 and December 2018 at a tertiary care center in Thailand and to identify associated factors of congenital syphilis. Our hypothesis was infants born to mothers who received no or scarce prenatal care and/or received no or inadequate syphilis treatment were likely to have congenital syphilis.

## Methods

We conducted a retrospective hospital-based case–control study in level 2 and 3 neonatal intensive care units (NICUs) with 33 beds at Vajira Hospital, Faculty of Medicine, Navamindradhiraj University. Vajira Hospital is a teaching university hospital with an in-patient capacity of 900 beds and 2000 deliveries per year. Inclusion criteria were neonates born alive to mothers with a reactive non-treponemal test for syphilis Rapid Plasma Reagin (RPR) during pregnancy or at delivery, between January 1, 2011 and December 31, 2018. The sample size was determined by considering 2 sample comparison of proportions for case–control study [15]. Among all the factors considered to be associated with congenital syphilis, no antenatal care (ANC) was considered to be the most important factor. We therefore chose this factor for sample size calculation. From previous study, the proportion of mothers who did not have ANC among infants with congenital syphilis (case) was 29.4% (p[exposure|case] = 0.294), while the proportion of mothers who did not have ANC among infants without congenital syphilis (control) was 6.2% (p[exposure|control] = 0.062). We considered the odds ratio of infants diagnosed with congenital syphilis among mothers with no ANC to be 2, with 95% level of confidence and 80% power and a ratio of case and control group 1:2 was used (r = 2). The final sample size after continuity correction became 45 for cases and 90 for controls. However, we included all the patients who met the inclusion criteria and were born between January 1, 2011 and December 31, 2018. Medical records of both mothers and neonates were reviewed by 2 pediatricians to categorize neonates according to the case definition of congenital syphilis. Data collection included maternal age, race, education, gestational age (GA), gravida, parity, number of ANC visits, GA when received first syphilis treatment, number of syphilis treatment given, duration from last syphilis treatment till birth, RPR titers before and after treatment, and route of delivery. Neonates' birth weight, APGAR scores, physical examination findings, laboratory results, radiologic findings, and in-hospital mortality were also collected from hospital medical records.

The study protocol was approved by the Vajira Institutional Review Board (IRB) of the Faculty of Medicine, Vajira Hospital, Navamindradhiraj University. Informed consent was waived by the IRB because the study was retrospective. Studies are reported according to the Observational Routinely collected health Data (RECORD) and Strengthening the Reporting of Observational Studies in Epidemiology (STROBE) Statements [16, 17, 18].

### Laboratory test for syphilis

Since 2015, our institution has used a reverse sequence algorithm for syphilis screening according to the CDC recommendations and Thai National Guideline 2015 [14, 19]. In this algorithm, chemiluminescent immunoassay (Treponema Pallidum Haemagglutination Assay [TPHA]) is used as treponemal test and then positive samples are confirmed by RPR. The test is done during the first ANC visit and in the third trimester. If the mother has not received prenatal care prior to delivery, the test is performed at the time she presents for delivery. A mother who has tested positive for syphilis receives additional care at Vajira Sexually Transmitted Disease clinic.

### Treatment of syphilis during pregnancy

Adequate treatment is defined as when all the following criteria are met: (1) Mother was given at least 1 dose (in early syphilis) and 3 doses (in late syphilis) of 2.4 million units of Benzathine penicillin G intramuscularly; (2) the period between treatment and delivery was >4 weeks apart from the last dose; (3) no evidence of recurrent infection. Recurrent infection is defined as a 4-fold increase in RPR after cure of disease (4-fold decreased or undetected antibody) [20]. Any treatment other than adequate treatment is defined as inadequate treatment.

### Cass definition of congenital syphilis

Diagnosis of congenital syphilis was based on the 2015 CDC surveillance case definition for congenital syphilis, Thailand National Guideline on the Management for the elimination of congenital syphilis 2015 with some modifications according to the American Academy of Pediatrics (AAP) Congenital syphilis 2018 [14, 19, 21].

Confirmed congenital syphilis is defined as a case that is laboratory confirmed with one of the following: “1. Non-treponemal test results in neonates ≥ fourfold compared to mother's 2.1 Direct identification of *T. pallidum* by specimen from the placenta, umbilical cord, secretions or lesions from infants using darkfield or fluorescent antibody test 2.2 Pathologic findings from placenta or umbilical cord consistent with congenital syphilis.”

Probable congenital syphilis is defined as a neonate or child who has a reactive non-treponemal test for syphilis and any one of the following: (1) Any evidence of congenital syphilis on physical examination; (2) any evidence of congenital syphilis on radiographs of long bones; (3) a reactive cerebrospinal fluid (CSF) venereal disease research laboratory (VDRL) test; and (4) in a non-traumatic lumbar puncture, an elevated CSF white blood cell (WBC) count >15 cells/mm^3^ during the first 30 d of life or >5 cells/mm^3^ after the first 30 d of life or protein >120 mg/dL during the first 30 d of life or >40 mg/dL after the first 30 d of life without other cause, regardless of CSF serology.

Possible congenital syphilis is a neonate who had normal physical examination, reactive non-treponemal test <4-fold the maternal titer and normal additional evaluations, and whose mother had untreated or inadequately treated syphilis at delivery.

Congenital syphilis less likely is a neonate who has normal physical examination and reactive non-treponemal test <4-fold the maternal titer, and whose mother received adequate treatment >4 weeks before delivery.

Neonates with confirmed and probable congenital syphilis as well as infants with possible congenital syphilis received additional evaluations, which include blood RPR titers, lumbar puncture (CSF cell counts, proteins and CSF VDRL), and radiograph of long bones. We defined CSF as a non-traumatic tap when the red blood cell count was <400 cells/mm. [3, 22], and the CSF specimen was sent out to an outside laboratory center for VDRL test. Hearing screen (otoacoustic emission test) was performed in all neonates as a part of universal hearing screen prior to discharge. Other tests including liver function tests and cranial ultrasound were performed if indicated.

Neonates with confirmed and probable congenital syphilis as well as neonates with possible congenital syphilis received aqueous crystalline penicillin at G 50,000 U/kg intravenously every 12 h (<7 d old) or every 8 h (≥7 d old) for 10 d. Neonates with congenital syphilis less likely were treated with a single dose of intramuscular benzathine penicillin G in accord with the AAP and CDC guidelines [20, 21].

We included confirmed and probable congenital syphilis as cases of congenital syphilis defined as “case group” and infants not categorized as above were included in the “control group.”

Baseline characteristics were reported as frequencies and percentages for categorical variables. Continuous variables were reported as means and standard deviations or median and interquartile range (IQR) as appropriate. We estimated the incidence of congenital syphilis by dividing the number of congenital syphilis that met the CDC surveillance case definition by the number of total live births during the study period. Factors associated with congenital syphilis were analyzed using univariable analysis and variables with *P* < 0.05 were analyzed in multiple logistic regression analysis. A *P*-value <0.05 was considered as statistically significant. Data were analyzed using IBM SPSS Statistics V28.0.

## Results

During January 1, 2011 and December 31, 2018, there were 19,558 live births at Vajira hospital, with 134 neonates born to mothers with positive syphilis screening. Eight neonates were excluded due to; missing records in 6 cases, the mother left the hospital against medical advice in 1 case, and 1 neonate was transferred out to another hospital. None of the infants were born to women who never had syphilis serology, since it is standard to perform serology screening even in mothers who had no prenatal care or serology screening before delivery when admitted to the labor room or postpartum ward. Among 126 neonates with maternal syphilis, 40 neonates met the criteria of congenital syphilis: 1 neonate (0.79%) who had RPR titer 4-fold higher than the mother was diagnosed as confirmed congenital syphilis and the remaining 39 neonates was categorized as probable congenital syphilis. The mother to infant transmission rate was 31.7% (95% confidence interval [CI]: 23.7–40.6). The incidence of congenital syphilis was 204 per 100,000 live births (95% CI: 146–278). The total live births, number of neonates born to mothers with syphilis, and neonates diagnosed as congenital syphilis by year of birth are shown in **Table 1**. The proportion of neonates born to mother with syphilis was 0.2% in 2011, increased to 1.6% in 2017, and was 1.3% in 2018. The maternal and neonatal characteristics are shown in **Table 2**.

**Table 1.** Total live births, neonates born to mothers with syphilis and number of neonates diagnosed with congenital syphilis by year of birth.

**Year of birth**	**Total live births**	**Number of neonates born to mothers with syphilis (%)**	**Neonates diagnosed with congenital syphilis^†^**	**Rate of congenital syphilis transmission (%)**	**Incidence of congenital syphilis per 100,000 live births**
2011	3,070	6 (0.2)	2	33.3	65.14
2012	2,945	4 (1.3)	1	25.0	33.95
2013	2,664	6 (0.2)	2	33.3	75.08
2014	2,534	8 (0.3)	5	62.5	197.32
2015	2,154	9 (0.4)	6	66.7	278.55
2016	2,115	33 (1.5)	9	27.3	425.53
2017	2,064	34 (1.6)	7	20.5	339.14
2018	2,012	26 (1.3)	8	30.8	397.61
Total	19,558	126 (0.6)	40	31.7	204.52

† Congenital syphilis includes confirmed and probable congenital syphilis

**Table 2.** Maternal and neonatal characteristics of 126 neonates born to mothers with syphilis.

**Characteristics**	**Case (N = 40)**	**Control (N = 86)**	**Total (N = 126)**

	**n (%)**	**n (%)**	**n (%)**
Maternal characteristics			
Age (years)			
<20	17 (42.5)	30 (34.9)	47 (37.3)
20–34	19 (47.5)	45 (52.3)	64 (50.8)
≥35	4 (10.0)	11 (12.8)	15 (11.9)
Median (IQR)	20 (18–22.5)	21 (18–25)	20 (18–24)
Ethnicity			
Thai	37 (92.5)	82 (95.3)	119 (94.4)
Others	3 (7.5)	4 (4.7)	7 (5.6)
Education (n = 60)			
Primary school	3 (33.3)	16 (31.4)	19 (31.7)
Secondary school	6 (66.7)	33 (64.7)	39 (65.0)
Bachelor degree or higher	0 (0.0)	2 (3.9)	2 (3.3)
Maternal co-infection			
HIV	2 (5.0)	2 (2.3)	4 (3.2)
Hepatitis B	1 (2.5)	1 (1.2)	2 (1.6)
Antenatal care†			
Yes	36 (90.0)	79 (91.9)	115 (91.3)
No	4 (10.0)	7 (8.1)	11 (8.7)
Maternal treatment			
Adequate	14 (35.0)	51 (59.3)	65 (51.6)
Inadequate	13 (32.5)	17 (19.8)	30 (23.8)
No treatment	13 (32.5)	18 (20.9)	31 (24.6)
Mode of delivery			
Vaginal	31 (77.5)	57 (66.3)	88 (69.8)
Cesarean section	9 (22.5)	26 (30.2)	35 (27.8)
Vacuum extraction	0 (0.0)	3 (3.5)	3 (2.4)
Neonatal characteristics			
Sex: Male	22 (55.0)	39 (45.3)	61 (48.4)
Gestational age (weeks)			
<37	13 (32.5)	10 (11.6)	24 (19.0)
37–42	27 (67.5)	74 (86.0)	100 (79.4)
>42	0 (0.0)	2 (2.3)	2 (1.6)
mean ± SD	37.05 ± 2.81	38.02 ± 2.36	37.73 ± 2.55
Birth weight (grams)			
<1000	1 (2.5%)	0 (0.0%)	1 (0.8%)
1001–1500	1 (2.5%)	1 (1.2%)	2 (1.6%)
1501–2000	3 (7.5%)	4 (4.7%)	7 (5.6%)
2001–2500	7 (17.5%)	8 (9.3%)	15 (11.9%)
>2500	28 (70.0%)	73 (84.9%)	101 (80.2)
mean ± SD	2753.98 ± 680.87	2882.78 ± 490.16	2841.89 ± 558.24
Low birth weight (<2500 grams)			
Yes	12 (30.0%)	13 (15.1%)	25 (19.8%)
No	28 (70.0%)	73 (84.9%)	101 (80.2%)
Birth weight for GA			
SGA	2 (5.0%)	3 (3.5%)	5 (4.0%)
AGA	37 (92.5%)	81 (94.2%)	118 (93.7%)
LGA	1 (2.5%)	2 (2.3%)	3 (2.4%)
Birth asphyxia‡			
No	34 (85.5%)	78 (90.7%)	112 (88.9%)
Yes	6 (15.0%)	8 (9.3%)	14 (11.1%)

† Antenatal care is defined as at least 1 visit of antenatal care.

‡ Birth asphyxia is defined as Apgar score ≤7 at 5 minutes of life.

IQR, interquartile range; SD, standard deviation; GA, gestational age; SGA, small for gestational age; AGA, appropriate for gestational age; LGA, large for gestational age.

Most of the mothers with syphilis were of Thai ethnicity and 91.3% had attended ANC. Their median age was 20 years old (IQR 18–24) and 37.3% were teenage mothers (<20 years old), with 42.5% and 34.9% in the case and control group, respectively. There were 61 neonates (61/126, 48.4%) whose mothers had inadequate syphilis treatment or no treatment. The proportion of preterm birth and low birth weight in the case group were 32.5% and 30.0%, respectively, versus 11.6% and 15.1%, respectively, in the control group.

Clinical characteristics, radiologic, laboratory, and CSF findings of neonates with congenital syphilis are shown in **Table 3**. Fourteen neonates (35.0%) had abnormal physical findings, in which the 2 most common findings were skin desquamation and hepatosplenomegaly in 17.5% and 10.0%, respectively. Six out of 32 neonates (18.8%) with available radiological study results had abnormal findings compatible with congenital syphilis. Lumbar puncture was performed in 38 neonates. Three infants had non-traumatic taps. Among 35 infants with non-traumatic taps, there were 30 cases with abnormal findings (85.7%), including pleocytosis or elevated protein, out of which 2 cases had positive CSF VDRL (**Table 3**). One neonate with positive CSF VDRL had repeated CSF examination at 26 d of age, which revealed non-reactive CSF VDRL. The other neonate had repeated CSF VDRL twice at 7 d and 32 d of life and both specimens revealed non-reactive VDRL results.

**Table 3.** Clinical, radiologic, complete blood count and CSF findings of 40 neonates with congenital syphilis†

**Findings**	**n (%)**
Abnormal physical examinations^‡^ (14/40 cases=35.0%)	
Skin desquamation	7 (17.5%)
Hepatosplenomegaly	4 (10.0%)
Rash	3 (7.5%)
Petechiae	2 (5%)
Abnormal radiologic findings^§^ (6/32 cases=18.8%)	
Cortical erosion	1 (3.1%)
Osteolytic lesion	1 (3.1%)
Radiolucent line at metaphyseal plate (osteitis)	5 (15.6%)
Abnormal complete blood counts (7/40 cases=17.5%)	
Anemia (Hematocrit < 40%)	2 (5 %)
Thrombocytopenia (Platelet < 150,000)	7 (17.5%)
Abnormal CSF fluid examinations^||^ (30/35 cases= 85.7%)	
CSF Pleocytosis (CSF WBC > 15 cells/mm^3^)	13 (37.1%)
Elevated CSF protein (CSF protein > 120 mg/dL)	20 (57.1%)
Positive CSF VDRL	2 (5.7%)

† Congenital syphilis includes confirmed and probable congenital syphilis.

‡ One infant may have more than 1 positive findings.

§ One infant had both osteolytic and radiolucent line.

|| Four infants had both pleocytosis and elevated protein, one infant had both elevated protein and positive.

VDRL CSF, cerebrospinal fluid; WBC, white blood cell; VDRL, venereal disease research laboratory test.

Among 126 neonates, 3 (2.8%) died. All of them were in the control group. The cause of death in 2 neonates was persistent pulmonary hypertension of the newborn. The third neonate died of cardiac tamponade.

By univariable analysis, factors associated with congenital syphilis were maternal RPR titers of >1:8 before delivery (most recent RPR titers) (odd ratio [OR]: 2.46, 95% CI: 1.11–5.49, *P* = 0.027), inadequate treatment or no treatment of maternal syphilis (OR: 2.79, 95% CI: 1.10–7.08, *P* = 0.031 and OR: 2.63, 95% CI: 1.04–6.65, *P* = 0.041, respectively), and preterm birth (OR: 3.66, 95% CI: 1.44–9.31, *P* = 0.006). When using multivariable analysis, inadequate maternal treatment and preterm birth were associated with congenital syphilis (adjusted odd ratio [aOR]: 2.69, 95% CI: 1.02–7.11, *P* = 0.046 and aOR: 2.91; 95% CI: 1.01–8.39, *P* = 0.049, respectively) (**Table 4**).

**Table 4.** Univariable and multivariable analysis for factors associated with congenital syphilis in neonates born to mothers with syphilis.

**Characteristics**	**Case (N = 40)**	**Control (N = 86)**	**OR†**	**95% CI**	***P***	**OR‡**	**95% CI**	***P***
Maternal Age, years									
<20	19 (47.5%)	30 (24.9%)	1.00	Reference				
20–34	17 (42.5%)	45 (52.3%)	1.34	0.60–2.99	0.472			
≥35	4 (10.0%)	11 (12.8%)	0.86	0.24–3.05	0.817			
1st ANC visit									
1st trimester	10 (25%)	32 (37.2%)	1.00	Reference				
2nd or 3rd trimester	26 (65%)	47 (54.7%)	1.77	0.75–4.17	0.191			
No ANC	4 (10%)	7 (8.1%)	1.83	0.44–7.56	0.404			
Maternal RPR before delivery									
<1:8	14 (35.0%)	48 (55.8%)	1	Reference		1.0	Reference	
>1:8	23 (57.7%)	32 (37.2%)	2.46	1.11–5.49	0.027	2.01	0.87–4.68	0.105
Non-reactive	3 (7.5%)	6 (7.0%)	1.71	0.38–7.75	0.484	1.01	0.19–5.30	0.989
Maternal treatment									
Adequate	14 (35.0%)	51 (59.3%)	1.00	Reference		1.0	Reference	
Inadequate	13 (32.5%)	17 (19.8%)	2.79	1.10–7.08	0.031	2.69	1.02–7.11	0.046
No treatment	13 (32.5%)	18 (20.9%)	2.63	1.04–6.65	0.041	1.84	0.65–5.17	0.248
Mode of delivery									
Cesarean section/vacuum extraction	9 (22.5%)	29 (33.7%)	1.00	Reference				
Vaginal	31 (77.5%)	57 (66.3%)	1.75	0.74–4.17	0.204			
Preterm birth									
No	27 (67.5%)	76 (88.4%)	1.00	Reference		1.0	Reference	
Yes	13 (32.5%)	10 (11.6%)	3.66	1.44–9.31	0.006	2.91	1.01–8.39	0.049
Birth weight									
>2,500 g	28 (70%)	73 (84.9%)	1.00	Reference				
<2,500 g	12 (30%)	13 (15.1%)	2.41	0.98–5.90	0.56			

† Crude OR estimated by binary logistic regression.

‡ Adjusted OR estimated by multiple logistic regression adjusted for RPR, maternal treatment and preterm birth.

OR, Odds Ratio; CI, confidence interval; ANC, antenatal care; RPR, rapid plasma reagin.

## Discussion

The incidence of congenital syphilis at our institution between 2011 and 2018 was 204 per 100,000 live births (95% CI: 146–278). Among 40 cases, 1 was diagnosed as confirmed congenital syphilis and 39 cases were diagnosed as probable congenital syphilis. Cases of syphilis at our institution, increased in 2019–2021. There were 58 cases (confirmed and probable congenital syphilis), which is quite high compared with the previous years in 2011–2018. Factors associated with congenital syphilis were inadequate maternal syphilis treatment and preterm birth. Mothers with syphilis were mostly of Thai ethnicity, and up to almost 40% were teenage mothers (<20 years old). This is consistent with the findings of increased incidence of syphilis in teenage mothers in Thailand [12, 13]. The median age of mothers with syphilis from this study was 20 years old. This was quite similar to a previous study in Thailand, which reported the median (IQR) maternal age of 21 (18–32) years among 69 pregnant women [23], but was lower than the age of mothers with syphilis from other studies. A study from China reported 155 mothers with syphilis, of whom none were under 20 years old [10], as well as a report of 149 mothers with syphilis from Brazil, whose median age was 24 years old [7]. Despite the efforts from national campaigns to eliminate congenital syphilis in Thailand and the high proportion of mothers that attended ANC in our study, mothers of 48.4% of neonates (61/126) had no syphilis treatment or inadequate treatment, which was comparable to 41% among 69 pregnant women reported in other studies [23].

The clinical manifestations, radiologic findings, and complete blood counts of our cases were consistent with other reports [24, 25]. The CSF findings in our cases were abnormal in 85.7% (30/35), which was quite high. A report of CSF findings in 11 neonates with probable congenital syphilis found abnormal values in 4 cases (36.4%), which included 3 with elevated WBCs or protein and 1 with reactive RPR [23]. Because of the wide range of normal values for CSF protein, red blood cells, and WBCs in the neonatal period, it has been difficult to define the proportion of neonates with congenital syphilis who have abnormalities of these laboratory values. There is other consensus that identifies an abnormal CSF WBC count in neonates being evaluated for possible congenital syphilis as >25 cells/mm^3^ and protein as >150 mg/dL (>170 mg/dL if neonate is premature) [26], which is different from the CDC guidelines used in our study [14]. However, neonates with abnormal CSF laboratory values in our study had normal central nervous system (CNS) exam without any CNS symptoms (drowsiness, irritability, or leptomeningeal symptoms). Furthermore, a reactive CSF VDRL or RPR in neonate is considered to be less specific than in older children and adults, because the maternal non-treponemal IgG antibodies can pass from maternal serum to fetal and neonatal serum and diffuse into the CSF [26]. Although, per 2021 CDC recommendation, lumbar puncture is not necessary in neonates who complete a 10-d course of parenteral therapy, it still might be useful to document CSF abnormalities that would indicate the need for close follow-up [27].

The incidence of congenital syphilis at our center was 204 cases per 100,000 live births, while the incidence at other university hospitals in Thailand was 115 cases per 100,000 live births over the period from 2013 to 2017 [23], both of which were high when compared with Thailand's national data of 40 cases per 100,000 livebirths in 2018 [13]. Variations in the incidence of congenital syphilis in different settings were also reported from other countries, such as Brazil, which reported a congenital syphilis case rate between 2010 and 2015 of 490 cases per 100,000 live births in the northeast region and 283 cases per 100,000 live births in the Midwest region [28]. This difference in the case rate might depend on the different methods used in case ascertainment, difference in national guideline of timing of screening, the epidemiologic surveillance system at the national level which needed technical and operational conditions for the reporting staff, and the difference in socioeconomic circumstances and cultural characteristics of the population. Our institution is a referral center, so we might have more patients with complicated conditions referred to our center. Furthermore, the surveillance case definition for congenital syphilis may vary between and within countries and regions, because a globally accepted case definition does not yet exist [29]. Consistent use of a well-defined case definition allows trends in disease in a country to be monitored over time.

Factors associated with congenital syphilis in our settings were preterm birth and inadequate treatment of maternal syphilis, which were consistent with other reports [6, 7, 8, 9, 10, 31]. Preterm birth could be the cause for inadequate treatment in mother with late diagnosis of syphilis, while it could be the consequence of syphilis infection in the mother [1, 24, 30].

Early diagnosis and timely treatment of syphilis in pregnancy should prevent preterm birth and congenital syphilis. Further study to find reasons of inadequate or no maternal treatment is also needed. Continued monitoring of incidence of congenital syphilis at each center apart from the available national surveillance system will help to evaluate and improve patient care quality in the individual setting.

Our study has several limitations. First, we did not include stillbirths that met the definition of congenital syphilis, so the incidence of congenital syphilis could be somewhat underestimated. Second, we did not have the information on paternal serostatus, which might affect maternal treatment outcomes. Finally, we were not able to evaluate the long-term outcomes of these cases due to loss to follow-up. Despite these limitations, our study provides the update on the increase in incidence of congenital syphilis over an 8-year period at a referral care center in Thailand by using a consistent case definition.

## Conclusions

Our study adds to the limited data on the recent increase in incidence of congenital syphilis and associated factors at a single referral center in Thailand. There is a need for interventions to improve the prenatal care quality addressing the prevention of congenital syphilis with strategies to solve the problem of inadequate and no maternal treatment. Evaluation of prevention of congenital syphilis should also be done at other centers apart from the national surveillance system to ensure the successful elimination of congenital syphilis.
